# Remodelling of Cortical Actin Where Lytic Granules Dock at Natural Killer Cell Immune Synapses Revealed by Super-Resolution Microscopy

**DOI:** 10.1371/journal.pbio.1001152

**Published:** 2011-09-13

**Authors:** Alice C. N. Brown, Stephane Oddos, Ian M. Dobbie, Juha-Matti Alakoskela, Richard M. Parton, Philipp Eissmann, Mark A. A. Neil, Christopher Dunsby, Paul M. W. French, Ilan Davis, Daniel M. Davis

**Affiliations:** 1 Division of Cell and Molecular Biology, Imperial College London, London, United Kingdom; 2 Department of Biochemistry, University of Oxford, Oxford, United Kingdom; 3 Department of Physics, Imperial College London, London, United Kingdom; National Jewish Medical and Research Center/Howard Hughes Medical Institute, United States of America

## Abstract

Natural Killer (NK) cells are innate immune cells that secrete lytic granules to directly kill virus-infected or transformed cells across an immune synapse. However, a major gap in understanding this process is in establishing how lytic granules pass through the mesh of cortical actin known to underlie the NK cell membrane. Research has been hampered by the resolution of conventional light microscopy, which is too low to resolve cortical actin during lytic granule secretion. Here we use two high-resolution imaging techniques to probe the synaptic organisation of NK cell receptors and filamentous (F)-actin. A combination of optical tweezers and live cell confocal microscopy reveals that microclusters of NKG2D assemble into a ring-shaped structure at the centre of intercellular synapses, where Vav1 and Grb2 also accumulate. Within this ring-shaped organisation of NK cell proteins, lytic granules accumulate for secretion. Using 3D-structured illumination microscopy (3D-SIM) to gain super-resolution of ∼100 nm, cortical actin was detected in a central region of the NK cell synapse irrespective of whether activating or inhibitory signals dominate. Strikingly, the periodicity of the cortical actin mesh increased in specific domains at the synapse when the NK cell was activated. Two-colour super-resolution imaging revealed that lytic granules docked precisely in these domains which were also proximal to where the microtubule-organising centre (MTOC) polarised. Together, these data demonstrate that remodelling of the cortical actin mesh occurs at the central region of the cytolytic NK cell immune synapse. This is likely to occur for other types of cell secretion and also emphasises the importance of emerging super-resolution imaging technology for revealing new biology.

## Abstract 2

Super-resolution 3D imaging reveals remodeling of the cortical actin meshwork at the natural killer cell immune synapse, which is likely to be important for secretion of lytic granules.

## Author Summary

Natural Killer (NK) cells are immune cells that can recognise and kill virus-infected and cancerous cells. This killing requires an intercellular contact —termed an immune synapse—between the NK cell and its target cell through which molecules can be delivered to trigger lysis. Reorganisation of the NK cell cytoskeleton is essential for the delivery and release at the synapse of granules containing the cytolytic molecules. Understanding precisely how the cytoskeleton is involved in these cytolytic events has been hampered by our inability to resolve cytoskeletal structure at immune synapses by conventional light microscopy. Very recent advances in imaging technology have now provided the resolving power to see previously undetectable cellular structures. Here, we have used 3D super-resolution imaging to observe the structure of the actin cytoskeleton at the NK immune synapse. We found that a dense mesh of actin underlies the immune synapse and that it is remodelled upon NK cell activation. Domains within the actin meshwork open up specifying where the lytic granules dock and also where the microtubule-organising centre moves towards. Thus, actin remodelling occurs at the immune synapse during secretion and this may be important for the regulation of lytic granule secretion.

## Introduction

Natural Killer (NK) cells are lymphocytes of the innate immune system that protect against viral infection and tumour progression via contact-dependent cellular cytotoxicity and the release of immune mediators such as cytokines [1]. NK cell cytotoxicity involves the direct killing of virus-infected or tumour cells through the polarised release of cytolytic molecules from specialised secretory organelles called lytic granules [2]–[3]. To ensure that NK cell killing is only directed towards appropriate target cells, NK cell activation and lytic granule release are tightly regulated [4].

Activation of NK cells is regulated by a balance of activating and inhibitory signals through a multitude of germ-line encoded receptors which recognise ligands expressed on the surface of other cells [5]. Over the last decade, much research has studied how immune cell interactions, including NK cell interactions, are often accompanied by the segregation of proteins into micrometer- and submicrometer-scale domains at an immune synapse [6]–[9]. The balance of activating and inhibitory signals at the immune synapse is translated into an appropriate NK cell response [10]. If activating signals dominate, an activating or cytolytic synapse is assembled and downstream NK cell effector functions are triggered, such as lytic granule polarisation and directed secretion towards the target cell [11]–[16]. If inhibitory signals dominate, then an inhibitory synapse assembles in which the supramolecular assembly of proteins at the synapse signals for the cells to move apart [7],[17]. It is well established that actin polymerisation is important for degranulation [13], however how the temporal and spatial organisation of actin, as well as activating receptors, facilitates lytic granule secretion remains ill-defined.

In Cytotoxic T cells (CTLs) the organisation of the cytolytic synapse has been studied using protein-rich supported planar bilayers [18]. Microclusters of ligated T cell receptor (TCR) form at the synapse periphery and migrate centrally to form a mature cytolytic synapse in which the adhesion molecule LFA-1 accumulates in a peripheral ring around centrally clustered TCR [19]. The microtubule organising centre (MTOC) polarises to the synapse facilitating the delivery of lytic granules through a central secretory domain [20]–[22]. Although formation of a mature synapse is not always essential for cell lysis by T cells, it does increase the efficiency of lytic granule polarisation and target cell killing [23]–[24].

One of the best characterised activating receptors on NK cells, NKG2D, recognises “stress-inducible” ligands, such as MICA, which are upregulated following, for example, heat shock or UV-induced DNA damage [25]–[27]. Ligation of NKG2D at the synapse recruits downstream signalling molecules that activate pathways for cytoskeletal reorganisation and degranulation [28]–[29]. However, directed polarisation of lytic granules in primary NK cells also requires co-stimulation through the integrin LFA-1 [30]. Recent experiments in which NK cells were stimulated on protein-rich supported lipid bilayers have shown that LFA-1 and NKG2D are organised into a peripheral ring and granule secretion is directed through a central synaptic domain by a mechanism dependent on ligation of LFA-1 [31]. This important study gained high temporal and spatial resolution by replacing the target cell with a protein-rich supported lipid bilayer and using Total Internal Reflection Fluorescence (TIRF) microscopy.

Equivalent imaging of proteins at synapses between NK cells and target cells has not been achieved because conventional imaging methods using 3D image reconstruction from optical stacks cannot provide high enough speed or resolution to capture these events. To address this, we recently implemented a novel approach using optical tweezers which allowed high speed (>1 frame per second (fps)) and high resolution (∼250 nm) imaging of the intercellular immune synapse [32]. Here, we use this approach to follow the molecular reorganisation at a cytolytic synapse between an NK cell and target cell from the moment of intercellular contact. This reveals that microclusters of NKG2D and signalling molecules form and within a couple of minutes accumulate into a central ring-shaped structure that marks a border within which lytic granules dock.

Concurrent with receptor reorganisation, filamentous actin (F-actin) rapidly polymerises to form a dense ring at the synapse periphery [20],[33]. Blocking actin polymerisation with drugs inhibits NK cell cytotoxicity [34] and patients with mutations in the actin regulatory protein Wiskott-Aldrich Syndrome protein (WASp) have poor NK cell cytotoxic function [35]. However, polymerised actin may also create a barrier which polarised lytic granules need to traverse in order to dock and fuse with the plasma membrane. In CTLs it has been suggested this barrier is overcome by clearance of actin from the central region of the synapse where secretion is known to occur [20]. Imaging by diffraction-limited methods, such as confocal microscopy, suggested that actin also cleared from the centre of NK cell synapses [14],[33],[36]. However, recently it was demonstrated that secretion of polarised lytic granules in NK cells required the activity of the actin binding protein myosin IIA [37]–[38]. Specifically, lytic granules could still polarise when myosin IIA was inhibited or knocked down but degranulation was prevented. At first encounter, these different sets of data seem paradoxical: How can an actin-binding motor protein be critical for lytic granule secretion if F-actin has been entirely cleared from the synapse centre where the secretion occurs? This issue has been specifically discussed in-depth by Sanborn and Orange [39].

The root of this problem is that standard light microscopy does not have sufficient resolution to detect what happens to the cortical actin mesh at immune synapses. This is because light microscopy is limited by diffraction, resulting in a lateral resolution of ∼250 nm and axial resolution of ∼600 nm. Recent advances in a number of “super-resolution” microscopy techniques have enabled this diffraction barrier to be overcome [40]. This includes optical approaches such as structured illumination microscopy (SIM) and stimulated emission depletion microscopy (STED). Here 3D-structured illumination microscopy (3D-SIM) was used, which improves resolution by the reconstruction of multiple images produced with periodic illumination patterns in different phases and orientations. By 3D-SIM we acquired super-resolution images of F-actin, lytic granules, and the MTOC at the NK cell immune synapse with unprecedented sensitivity and spatial resolution. We demonstrate that F-actin forms a dense cortical mesh in resting human NK cells similar to that characterised in other secretory cell types [41]. When NK cells are activated through NKG2D, F-actin is still present within the centre of the cytolytic synapse but remodelling of the cortical actin mesh occurs precisely where the MTOC polarises and lytic granules accumulate.

## Results

### Dynamic Reorganisation of NKG2D and Signalling Molecules Grb2 and Vav-1 at Intercellular Cytolytic Synapses

**Figure 1:**
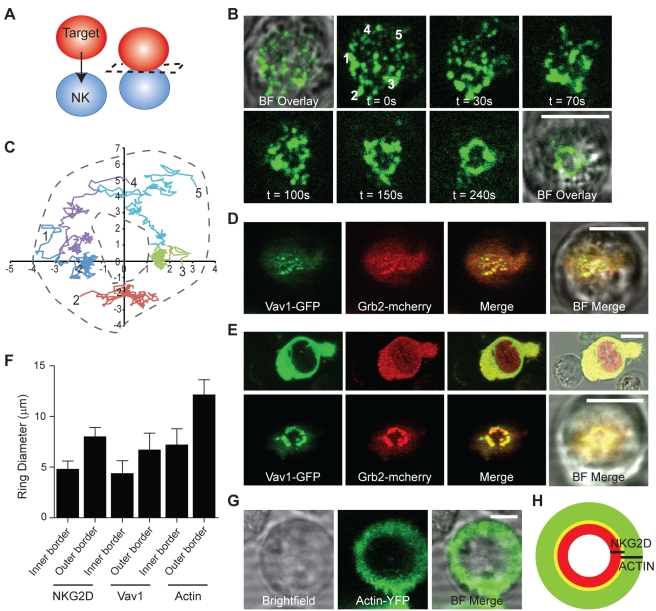
Organisation of NKG2D, Vav1, and Grb2 at the intercellular cytolytic NK cell synapse. (A) A schematic to show how optical tweezers were used to place a target cell into contact with an NK cell in order that the immune synapse could be imaged with higher resolution and at high speed. The dotted line shows the imaging plane. (B) Time-lapse imaging (1 fps) of NKG2D microcluster formation and supramolecular reorganisation into a ring-shaped structure between live conjugates of Daudi/MICA and NKL/NKG2D-GFP (representative of 67% of synapses, *n* = 45). (C) Tracks showing the movement of the individual NKG2D-GFP microclusters (labelled 1–5 in (C) between t = 0 and t = 150 s (the scale on the graph is in µm)). (D) Representative live cell image of a conjugate between Daudi/MICA and NKL transfected to express both Vav1-GFP and Grb2-mCherry, at the moment of cell-cell contact. (E) Representative live cell image of a conjugate between Daudi/MICA and NKL/Vav1-GFP/Grb2-mCherry. Upper panels show the conjugate immediately prior to reorientation using the optical tweezers (∼170 s after conjugate formation) and lower panels the same conjugate with the synapse orientated in the imaging plane. Vav1 and Grb2 co-localise, with Pearson's correlation coefficient R_r_ = 0.7, and organise into a central ring. (F) The diameter at the inner and outer borders of fluorescent protein rings imaged in NKL cells expressing either NKG2D-GFP, Vav1-GFP/Grb2-mCherry, or actin-YFP; graph shows mean ± SD (*n* = 10 cells). (G) Representative live cell image of a conjugate between Daudi/MICA and NKL expressing actin-YFP. (H) Scaled schematic representing the relative size and organisation of NKG2D-GFP and actin-YFP rings detected in NKL–Daudi/MICA conjugates. All scale bars = 10 µm.

Through recent improvements in imaging technology, it has now been established that within immune synapses, kinases, adaptors, and receptors accumulate within structures termed microclusters. T cell receptor signalling, for example, is initiated in such microclusters and these signals are terminated as microclusters move from the periphery to the central region of the synapse [42]–[44]. These early studies of microclusters used T cells interacting with protein-rich supported planar bilayers. Imaging of molecular events at an intercellular synapse can only be achieved by acquiring optical slices through a sample and then reconstructing the *“en face”* view of the synapse. This approach does not permit high speed imaging and, more critically, only provides micrometer-scale spatial resolution, which severely hampers detection of microclusters at intercellular synapses [45]. Recently microclusters have been seen in intercellular T cell synapses using optical tweezers combined with confocal microscopy [46]. Here, we used optical tweezers to trap a target cell and place it into contact with an NK cell such that the immune synapse can be imaged in the lateral imaging plane (Figure 1A). This facilitated high resolution time-lapse imaging of the immune synapse from initial NK cell-target cell contact through to formation of a mature cytolytic synapse.

To realise live cell imaging, we used the immortal human NK cell line NKL transfected to express NKG2D tagged with green fluorescent protein (NKL/NKG2D-GFP). For target cells, we used the B cell line Daudi, transfected to express MICA (Daudi/MICA), a ligand for NKG2D [47]. These target cells were efficiently lysed by NKL/NKG2D-GFP (Figure S1). This confirmed NKG2D-GFP as being functional since if NKG2D-GFP was not functional, it would act as a dominant negative and lysis would be impaired [48]. Additional evidence that the NKG2D-GFP construct was functional was that expression in Jurkat cells, which endogenously express DAP-10 but not NKG2D, allowed Jurkat to functionally respond to Daudi/MICA (unpublished data). Live cell imaging was then performed at a rate of 1 fps (Figure 1B, Video S1). Strikingly, NKG2D-GFP accumulated in distinct micrometer-scale clusters within a second after initial intercellular contact. Relatively large micrometer-scale clustering of NKG2D was absent at the surface of non-interacting cells (unpublished data), demonstrating that clustering was rapidly induced upon target cell recognition. Microclusters of NKG2D were observed to continuously form during the spreading of the NK cell over its target cell (0–45 s), and then moved into a stable central ring-shaped structure within 2–4 min of initial cell-cell contact (67% of cases; *n* = 50). At the limit of resolution, the ring-shaped organisation of NKG2D did not have any observable sub-structure. Tracking of NKG2D microclusters revealed that their trajectories were generally centripetal with a distribution of speeds ranging from 0.25 µm/s to 1.0 µm/s (Figure 1C).

In human NK cells, NKG2D forms a hexameric complex with the adaptor DAP-10 [27]. Upon NKG2D ligation, DAP-10 is phosphorylated and binds the scaffold protein Grb2 which in turn binds the signalling molecule Vav1 [49]. The recruitment of the Grb2-Vav1 signalling complex is an essential step in NKG2D-mediated cytotoxicity, as Vav1 activation triggers actin polymerisation pathways and degranulation [50]–[52]. In order to temporally resolve at which stage during immune synapse formation Grb2 and Vav1 signalling molecules are recruited to the immune synapse, NKL cell transfectants expressing Vav1-GFP and Grb2-mCherry were mixed with Daudi/MICA and were brought into contact using optical tweezers. Reminiscent of the supramolecular organisation of NKG2D, Vav1 and Grb2 microclusters formed immediately at the cell-cell interface (Figure 1D) and reorganised within ∼3 min into a central stable ring (Figure 1E, Figure S2A). Grb2 and Vav1 were consistently colocalised throughout synapse formation as determined by Pearson's correlation coefficients R_r_ = 0.3±0.09 (t = 0) and R_r_ = Rr = 0.7±0.1 (t = 170 s, n = 5). The accumulation of Grb2-mCherry and Vav1-GFP was dependent on the interaction between endogenous NKG2D and MICA, as neither molecule accumulated at the cell-cell interface with Daudi cells that do not express MICA (Figure S2B).

To determine the size and spatial relationship between different protein-rich domains, we compared the inner and outer diameters of the ring-shaped organisation of NKG2D-GFP or Vav1-GFP to the peripheral ring of actin (Figure 1F). For this, NKL transfected to express actin-YFP were conjugated to Daudi/MICA and imaged following reorientation of the conjugates with optical tweezers (Figure 1G). Rings of NKG2D-GFP and Vav1-GFP were similarly sized and fitted within the inner border of the peripheral actin ring at the NK cell synapse (schematically drawn to scale in Figure 1H). Taken together, these data show that at intercellular synapses microclusters of NKG2D and signalling molecules are recruited immediately upon conjugation and rapidly reorganise to form a ring-shaped structure at the synapse centre.

### Ring-Shaped Organisation of NKG2D Marks a Border for Lytic Granule Secretion

**Figure 2:**
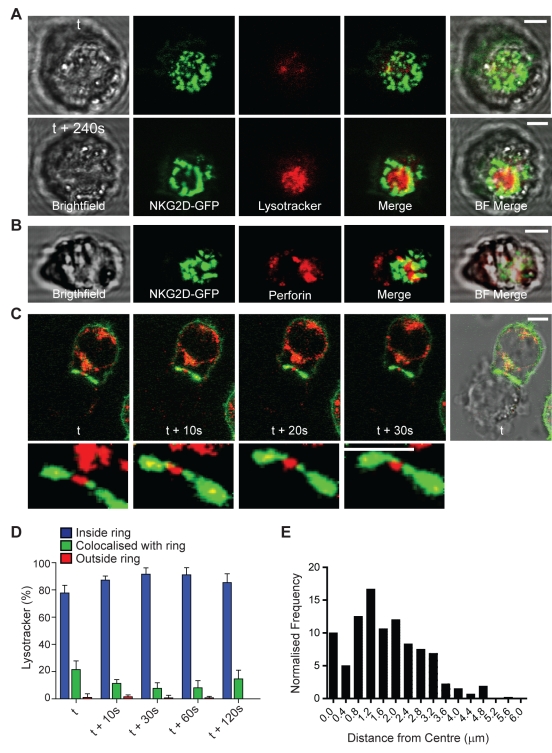
Lytic granules are delivered within the ring-shaped organisation of NKG2D at the synapse centre. (A) Time-lapse microscopy of live cell conjugates of NKL/NKG2D-GFP loaded with Lysotracker Red and Daudi/MICA. Lysotracker was absent from the early immune synapse when NKG2D microclusters initially formed (top, t) but appeared within the ring-shaped organisation of NKG2D at later time points (bottom, t+240 s) (*n* = 7). (B) Conjugates between NKL/NKG2D-GFP and Daudi/MICA were fixed and stained for perforin. Perforin (red) is detected within the central ring of NKG2D (green). (C) Live cell imaging shows vesicles marked with Lysotracker (red) move from the NKL to the target cell within regions in which NKG2D-GFP is less dense (green). Lower panels show the synapse between the NKL and target cell enlarged. (D) Quantification of the extent of Lysotracker staining localised within the inner border of the NKG2D ring, co-localising with the NKG2D ring or outside the outer border of the NKG2D ring. Graph shows mean ± SEM for granules from *n* = 10 cells analysed for 2 min post-NKG2D-GFP ring formation. (E) The distribution of the frequency normalised by area at a given radius from the NKG2D-GFP ring centroid of Lysotracker particles at the NK synapse. All scale bars = 5 µm.

To determine the relationship between the organisation of NKG2D and lytic granule secretion, NKL/NKG2D-GFP were loaded with Lysotracker Red, which marks acidic vesicles including lytic granules, and then individual cells were moved to contact Daudi/MICA using optical tweezers. Lysotracker was only accumulated in synapses where NKG2D had organised into a ring-shaped structure at the synapse. Before ring formation, when microclusters of NKG2D were present, Lysotracker had not yet polarised towards the synapse (Figure 2A, Video S2, Video S3, *n* = 24). As Lysotracker Red labels all acidic compartments within the cell we next set out to test that the vesicles localised within the NKG2D ring were indeed lytic granules. Conjugates formed between NKL/NKG2D-GFP and Daudi/MICA were fixed and immunostained for the specific lytic granule protein, perforin. Again, conjugates were imaged using optical tweezers to orientate the synapse to the imaging plane to realise high-resolution imaging. Polarised staining for perforin was consistently observed within the ring-shaped organisation of NKG2D (Figure 2B). However, it may be useful to note that imaging of perforin was hampered compared with live-cell imaging with Lysotracker, because this required using fixatives that perturb cell morphology and to some extent the organisation of NKG2D. For conjugates where Daudi/MICA were clearly undergoing apoptosis, as observed by extensive blebbing, granules marked by Lysotracker could be detected to dock at a site at the centre of the cell-cell interface not populated by NKG2D-GFP over a 30 s period (Figure 2C).

To assess the position of polarised Lysotracker relative to the ring-shaped organisation of NKG2D, the amount of Lysotracker at each synapse was compared for being (i) within the ring of NKG2D, (ii) colocalised with the ring, or (iii) outside the ring (Figure 2D). This showed that greater than 80% of Lysotracker staining was located within the ring of NKG2D at all time points analysed. Also, the distance of Lysotracker-stained vesicles or groups of vesicles from the centre of the NKG2D ring were quantified (Figure 2E, *n* = 10 cells). This clarified that Lysotracker-stained granules were distributed within a domain of radius ∼3.0 µm. The inner and outer edges of the NKG2D ring have diameters ∼5 µm and ∼8 µm, respectively. This is consistent with previous data using protein-rich lipid bilayers as surrogates for target cells, where a synaptic ring of NKG2D marked a secretory domain where lytic granules accumulated [31]. Thus, the ring-shaped organisation of NKG2D marks out a boundary for the primary location of granule secretion within which granules dock.

### Super-Resolution Imaging of NK Cell F-Actin by Structured Illumination Microscopy

A major limitation in determining the organisation and role of actin at the cytolytic synapse is the diffraction limited resolution of conventional microscopy techniques. In order to resolve the structure of F-actin at the immune synapse, we therefore used a super-resolution fluorescence imaging technique, 3D-Structured Illumination (SI) microscopy that can give a 2-fold improvement in resolution when compared to confocal microscopy [53]. Importantly, this is a super-resolution technique that can image away from the surface of the coverslip unlikeimaging methods that use TIRF microscopy for example [40].

**Figure 3:**
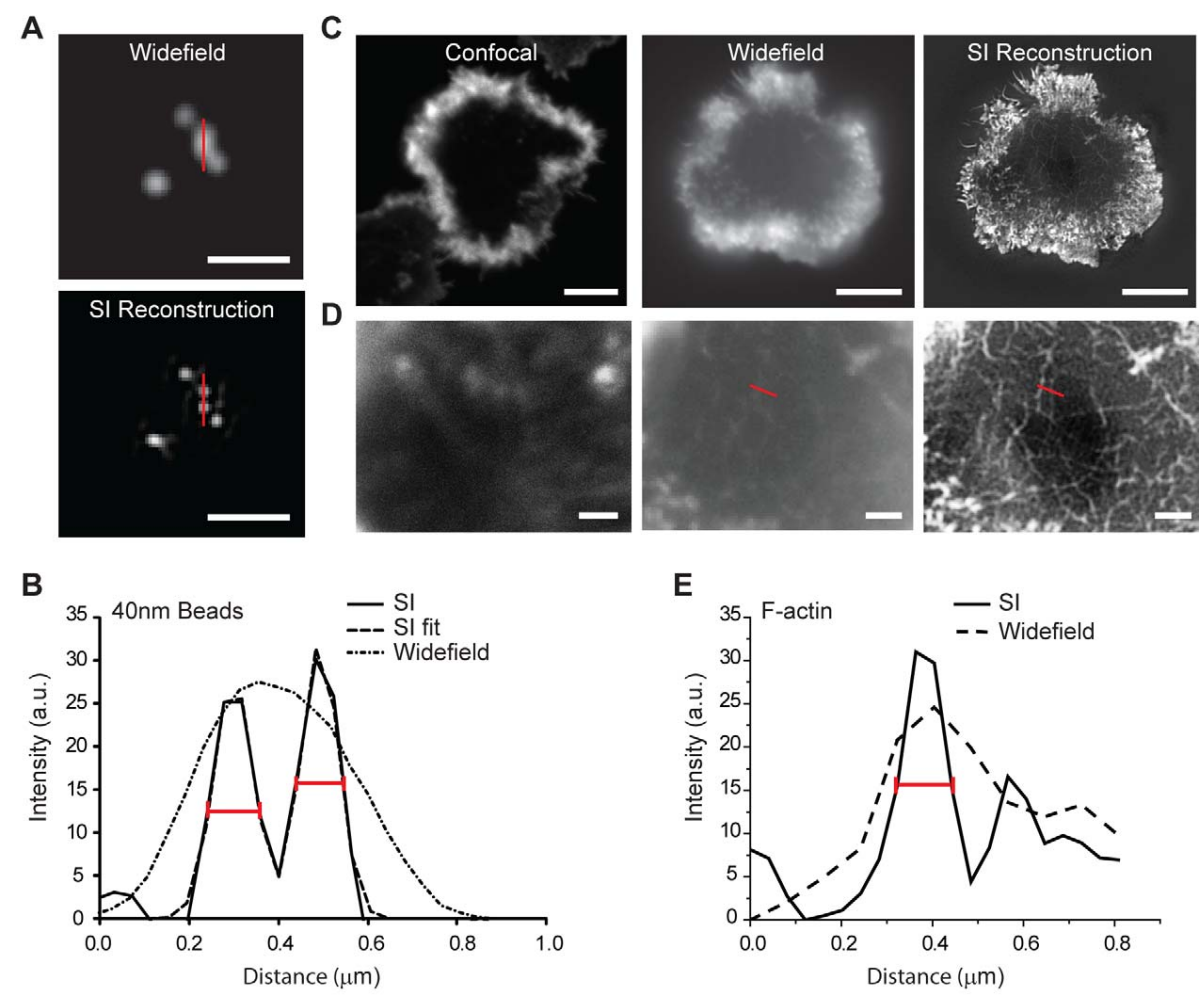
Structured Illumination microscopy permits super-resolution imaging of F-actin at the NK cell synapse. (A) Widefield (top) and SI images (bottom) of 40 nm beads. Bar = 1 µm. (B) Plot profiles from the region marked by red bars in (A) show that two beads approximately 200 nm apart can only be resolved following SI reconstruction. Fitting of the curve with two Gaussians gives full width half maximums (FWHMs, red bars on the graph) of 99 nm and 105 nm for each bead, respectively. (C) Confocal (left), widefield (middle), and SI image (right) of human primary NK (pNK) cells activated on a surface coated with anti-NKG2D mAb. Bars = 5 µm. (D) Regions at the centre of the synapses in (C) are enlarged to demonstrate the increased level of detail in cortical F-actin structure labelled using AlexaFluor488-conjugated phalloidin that can be observed when SI microscopy is used. Bars = 1 µm. (E) Plot profiles to directly compare the widefield and SI reconstructed image for the region indicated by the red bar shown in panel D. Estimation of the FWHM (red bar on the graph) from the Gaussian fit of the SI data gives a resolution of ∼115 nm.

We demonstrated the improvement in resolution in our SI setup by showing that 40 nm beads less than 200 nm apart can only be resolved following reconstruction of acquired SI widefield images (Figure 3A) and that resolution as determined by the FWHM for each bead was ∼100 nm (Figure 3B). We compared confocal, widefield, and SI images of F-actin stained with phalloidin in human primary NK (pNK) cells expanded in interleukin-2 (IL-2), activated to form a distinctive peripheral actin ring through ligation of NKG2D using monoclonal antibody-coated glass sides (Figure 3C). Crucially, the use of SI microscopy revealed the fine mesh of actin filaments at the centre of the synapse (Figure 3D). In these images, F-actin filaments <200 nm apart could be detected as individual structures with a resolution limit ∼110 nm (Figure 3E). SI microscopy therefore provides the ability to detect F-actin structures and F-actin structural changes at the NK cell synapse that could not previously be detected using either confocal or widefield imaging techniques.

### Cortical F-Actin Is Remodelled But Not Entirely Cleared from the NK Cell Cytolytic Synapse

**Figure 4:**
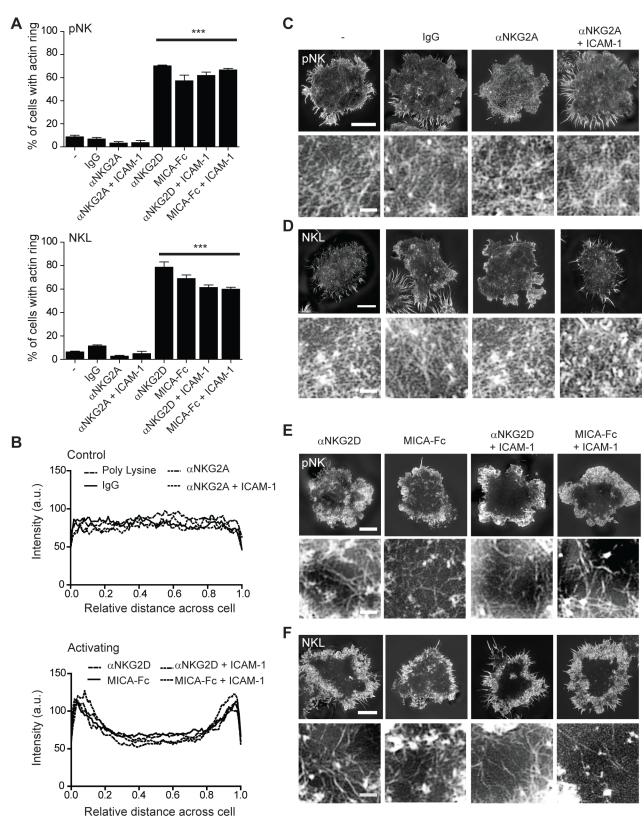
Organisation of cortical actin at the NK cell immune synapse. (A) The proportion of pNK cells (top) or NKL cells (bottom) that form a F-actin ring when stimulated on surfaces coated with control protein (poly-L-lysine, Isotype-matched mAb), inhibitory (αNKG2A, αNKG2A+ICAM-1), or activating mAb/ligand (αNKG2D, αNKG2D+ICAM-1, MICA-Fc, MICA-Fc+ICAM-1). Graph shows the mean ± SEM, *n*>200, *** *p*<0.001. (B) The intensity of F-actin distribution in SI microscopy images across pNK cells stimulated on control or inhibitory surfaces (top) or activating surfaces (bottom). Graphs show the mean, *n* = 10–75 cells per condition. (C) Representative images obtained by SI microscopy of cortical F-actin at the interface between pNK cells and coverslips coated with inhibitory or control ligands. Bar = 5 µm. The central synaptic region is enlarged in the lower panels. Bar = 1 µm. (D) SI images of cortical F-actin structure at the interface between NKL cells and surfaces coated as in (C). Bar = 5 µm. The centre of the synapse is enlarged in the lower panels. Bar = 1 µm. (E) SI images of cortical F-actin structure at the interface between pNK cells and surfaces coated with activating antibody or ligand. Bar = 5 µm. The central region of the synapse is enlarged in the lower panels. Bar = 1 µm. (F) SI images of cortical F-actin structure at the interface between NKL cells and surfaces coated as in (E). Bar = 5 µm. The central region of the synapse is enlarged in the lower panels, bar = 1 µm.

We and others have shown that when activating signals dominate over inhibitory signals NK cells stop migrating, spread symmetrically, and form a distinct peripheral ring of polymerised F-actin [13]–[14],[54]–[55]. Here, we first set out to clarify if ligation of NKG2D was sufficient to induce actin polymerisation in either pNK cells isolated from peripheral blood and expanded in IL-2, or in NKL cells. The percentage of cells that formed a peripheral ring of F-actin was determined following stimulation on glass surfaces coated with (3.0 µg/ml) mAb against NKG2D or (2.0 µg/ml) MICA-Fc, with or without co-stimulation of LFA-1 using (2.5 µg/ml) ICAM-1. We also compared ligation of the inhibitory receptor NKG2A using (3.0 µg/ml) mAb with or without co-ligation of LFA-1 (Figure 4A). IL-2 activated pNK or NKL, stimulated via NKG2D, but not NKG2A, formed a ring of polymerised F-actin at the periphery of the area in contact with the slide surface without the need for LFA-1 co-activation (Figure 4B). The dense peripheral ring of F-actin formed by NK cells on slides had the same inner and outer diameter to that measured in intercellular conjugates (Figure S3). IL-2 activated pNK and NKL did not form peripheral actin rings on uncoated surfaces or surfaces coated with control isotype-matched mAb. The response to immobilised anti-NKG2D mAb or MICA-Fc could also be blocked by the addition of excess anti-NKG2D mAb but not by addition of excess anti-CD16 mAb (Figure S4). Furthermore, NKL used in the study did not express the Fc receptor CD16 (determined by flow cytometry; unpublished data). This confirmed that stimulation through NKG2D can trigger the assembly of a ring of F-actin at the synapse periphery.

To compare the molecular organisation of actin at activated and inhibitory NK cell synapses, super-resolved imaging of cortical F-actin was carried out by SI microscopy for pNK and NKL. Cells were incubated on slides coated with anti-NKG2D mAb, anti-NKG2A mAb or MICA-Fc, with or without ICAM-1, or on poly-L-lysine or isotype-matched mAb as controls (Figure 4C–F). pNK cells have a dense mesh of cortical branching F-actin that was unchanged following ligation of the inhibitory receptor NKG2A (Figure 4C and 4D, lower panels). This may relate to previous observations that accumulation of some proteins at inhibitory NK cell synapses is largely an actin-independent process, at least with some target cells [7],[56]–[57]. In activated pNK and NKL cells, in which a peripheral ring of F-actin had formed, actin filaments could also clearly be detected throughout the synapse centre (Figure 4E and 4F). Organisation of actin in the synapse centre formed a much less dense mesh of branching actin in comparison to actin at the synapse periphery or at inhibitory synapses (Figure 4E and 4F, lower panels). Co-ligation of LFA-1 did not alter the extent of actin polymerisation or re-organisation. Thus, in contrast to previous work with lower resolution imaging [20],[33],[36], super-resolution images reveal that NK cell activation does not lead to the complete clearance of F-actin from the synapse centre.

### Parameterisation of the Organisation of F-Actin at the NK Cell Immune Synapse

**Figure 5:**
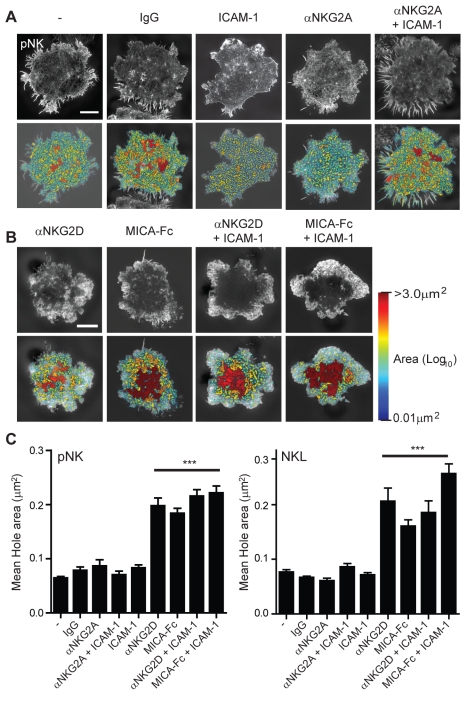
Increased periodicity of the cortical actin mesh at the centre of the NK cell synapse. (A) SI microscopy images of cortical F-actin in pNK cells stimulated on surfaces coated with control (poly-L-lysine, Isotype-matched mAb, ICAM-1) or inhibitory proteins (αNKG2A, αNKG2A + ICAM-1). Actin mesh domains (or “holes”) are shown as heat maps related to hole area, with the smallest holes shown in blue set at the resolution limit of the microscope (0.01 µm^2^) and largest areas (>3.0 µm^2^) shown in red. (B) Heat mapped SI images of cortical F-actin in pNK cells activated on surfaces coated with activating mAb/ligand (αNKG2D, αNKG2D+ICAM-1, MICA-Fc, MICA-Fc+ICAM-1). All scale bars = 5 µm. (C) Quantification of gaps or “holes” in the F-actin mesh in the synapse centre for pNK cells (top) or NKL cells (bottom) stimulated on control, inhibitory, or activating surfaces. The periodicity (and “hole area”) of the actin mesh significantly increases when cells are activated, with or without LFA-1 engagement. Graph shows mean ± SEM (*n* = 10–75 cells, *** *p*<0.001).

To measure the changes in the F-actin structure, we calculated the area of the gaps between individual F-actin filaments within the central synapse (termed here as “holes”), using a custom MatLab program (see Text S1). This analysis revealed regions in which the actin mesh structure had opened up across the pNK cell surface synapse (Figure 5A and 5B). Specifically, false-colour heat maps were used to reveal areas of holes at the synapse for the range of stimulatory or inhibitory conditions. When pNK or NKL cells were in contact with slides with either no stimulation, or coated with ICAM-1 alone or inhibitory anti-NKG2A mAb, the mean hole area within the central synapse was <0.07 µm^2^. However, the hole size dramatically increased to >0.2 µm^2^ when pNK or NKL cells were activated (Figure 5C). Co-stimulation through LFA-1 did not affect the size of holes in the actin mesh. The average width of holes between actin filaments within the synapse centre, calculated from the measured areas, increased from ∼200 nm to ∼450 nm following NK cell activation via NKG2D, an increase that is not resolvable by confocal microscopy.

### Predicted Regions of Lytic Granule Penetration at NK Cell Immune Synapses

**Figure 6:**
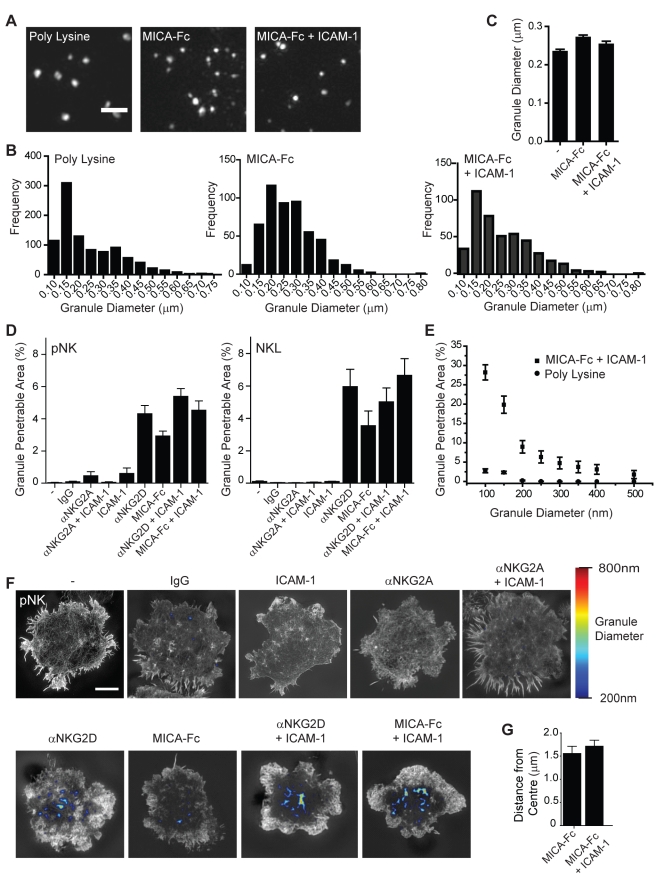
Predicted regions of lytic granule penetration across the NK cell immune synapse. (A) SI microscopy of lytic granules stained for perforin in pNK cells stimulated on control (poly-L-lysine, left) or activating (MICA-Fc, middle, or MICA-Fc + ICAM-1, right) surfaces. Bar = 1 µm. (B) Distributions of granule diameters above the measured 100 nm SI microscope resolution limit for pNK cells stimulated as in (A) (*n* = 10 cells per condition). (C) Average granule diameter for pNK cells stimulated as in (A) (mean ± SEM, *n*>400). (D) The fraction of the synapse predicted to be readily penetrable by a granule with a diameter of 250 nm quantified for pNK cells (left) or NKL cells (right) stimulated on control, inhibitory, or activating surfaces. Graphs show mean ± SEM, *n* = 10–75 cells. (E) Quantification of the proportion of the immune synapse in pNK on control surfaces or activated with MICA-Fc + ICAM-1 predicted to be penetrable by granules of diameters ranging from 100 nm to 500 nm. Graph shows the mean ± SEM (*n* = 10 cells). (F) Regions within the cortical F-actin mesh through which a lytic granule of diameter 200 nm (blue) to 800 nm (red) may penetrate were mapped on images of pNK cells stimulated on control, inhibitory, and activating surfaces. (G) The average distance of the predicted granule penetrable areas from the synapse centre was measured and graphs show mean ± SEM (*n* = 10 cells). Bar = 5 µm.

We next set out to test if the opening up of the cortical actin mesh by approximately 250 nm would be sufficient to permit lytic granules to traverse the cortical actin relatively unimpeded. This required determining the size of lytic granules. Thus, lytic granules were imaged by SI microscopy in unstimulated pNK cells or cells activated through ligation of NKG2D by MICA with or without co-stimulation of LFA-1 (Figure 6A). The diameters of all granules were calculated (Figure 6B), with the average granule diameter independent of NK stimulation being 251±2 nm (*n* = 2,014 granules from 40 cells) (Figure 6C). The distribution of granule diameters in primary NK cells was similar to granule sizes reported previously using electron microscopy of NK cell lines and cytotoxic T lymphocytes [58]. Significantly, these data indicated that an average change in the width of holes in the cortical actin structure from ∼200 nm to ∼450 nm might well be sufficient to allow vesicles of 250 nm diameter to pass through.

Thus we next calculated the percentage of the central synaptic region in which actin filaments were far enough apart to create a space that a granule of 250 nm in diameter could traverse, using a custom MatLab program (see Text S1). Briefly, to determine these regions within the SI images the MatLab program calculated a distance transform of the whole cell image by analysing the closest distance from an actin negative pixel or “hole” to an actin positive pixel or “filament.” This image was then thresholded by the granule radius to produce an image in which granule penetrable holes are those through which granules could penetrate without deforming. Importantly, analysis of pNK or NKL cells imaged under control, inhibitory, or activating conditions showed a granule with 250 nm diameter would rarely or never fit through the cortical F-actin mesh unless the NK cell was activated (Figure 6D). When NKG2D was ligated, using mAb or MICA-Fc, 4.5±0.25% of the synaptic region would be penetrable by a lytic granule of 250 nm unimpeded. This suggests that cortical actin may act as a barrier to lytic granule secretion, unless activating receptors are ligated which trigger the cortical actin mesh to open up. Even if granules were able to deform to only 100 nm in diameter, they would not easily pass through the cortical actin structure in unstimulated NK cells, i.e. 2.5±0.2% of the synapse would be penetrable (Figure 6E), whereas actin remodelling after NKG2D-mediated activation leads to 28.1±1.9% of the synapse being penetrable to a granule of diameter 100 nm. Thus, NK cell activation leads to a relatively small portion of the central synapse predicted to be easily penetrable by lytic granules.

We next mapped the location of regions predicted to be penetrable by a granule of between 200 nm and 800 nm, covering the range of granule diameters measured (Figure 6F). Interestingly, when cells were activated through NKG2D, with or without LFA-1 co-stimulation, the penetrable region of the interface was organised into a few distinct clusters within the central synapse rather than individual penetrable “holes” being distributed evenly across the interface. These penetrable domains were absent when NK cells were not stimulated or were inhibited, confirming that granules with diameter 200–800 nm would not be able to penetrate through NK cell cortical actin. The average distance of these domains from the synapse centre was 1.56±0.15 µm and 1.71±0.13 µm for cells stimulated by MICA-Fc or MICA-Fc and ICAM-1, respectively. This is similar to the average distance from the synapse centre for Lysotracker-stained vesicles or groups of vesicles seen at live intercellular synapses (Figure 2E, 2.37±0.13 µm). Taken together, these data indicate that lytic granules may only easily traverse the NK cell cortical actin structure in distinct central domains in which the periodicity of the cortical actin mesh is increased following NKG2D-mediated stimulation.

### Cortical F-Actin Rearrangements Can Be Stimulated through Engagement of CD16

**Figure 7:**
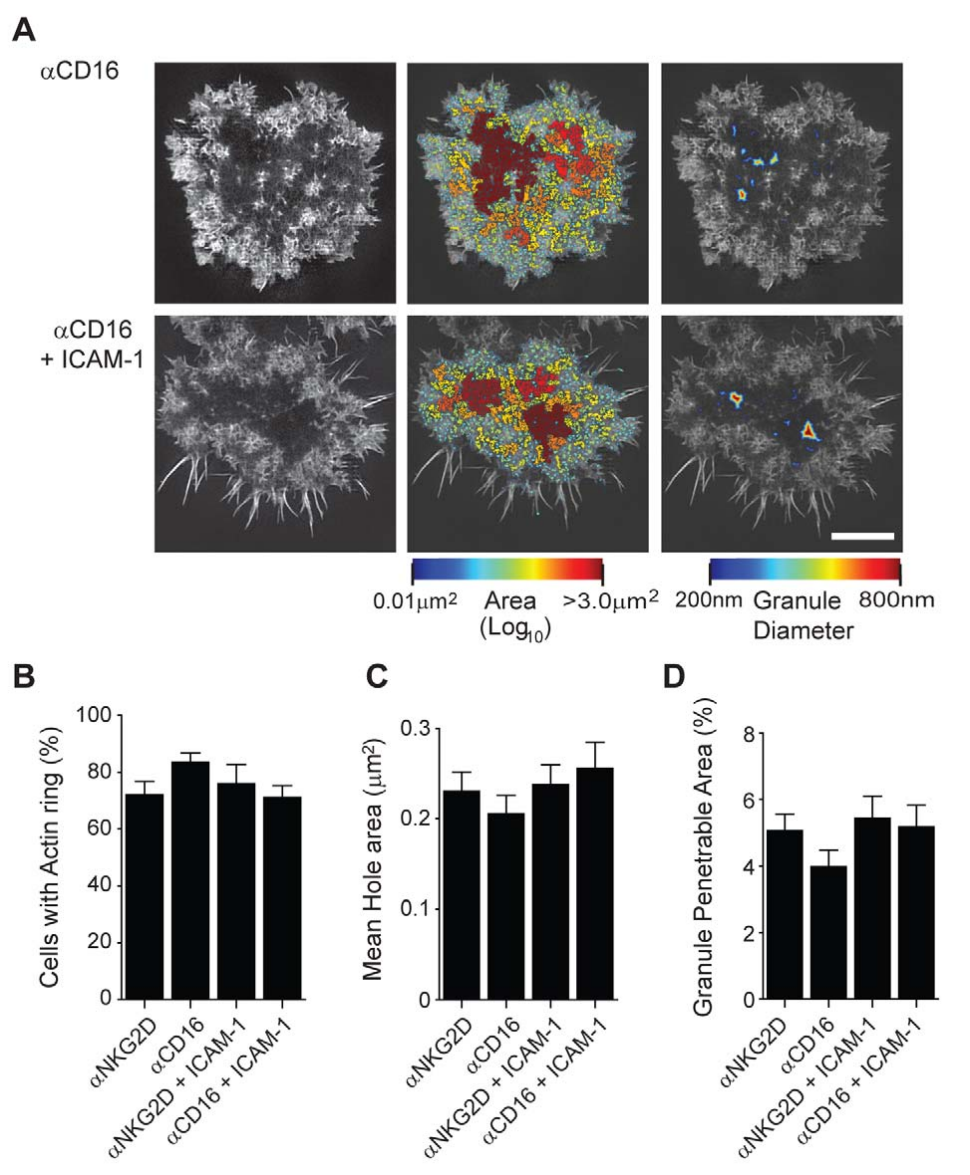
Cortical F-actin rearrangements can be stimulated through engagement of CD16. (A) Images from SI microscopy of cortical F-actin in pNK cells stimulated on surfaces coated with mAb to CD16 with or without ICAM-1. The central panel shows actin mesh domains as a heat map related to hole area with smallest holes shown in blue (0.01 µm^2^) and largest in red (>3.0 µm^2^). The right panel shows the regions within the cortical F-actin mesh through which a lytic granule of diameter 200 nm (blue) to 800 nm (red) may penetrate. Bar = 5 µm. (B) The proportion of pNK cells that form an F-actin ring when stimulated on surfaces coated with αNKG2D or αCD16 with or without ICAM-1. (C) The mean hole area within the central region of the pNK cell synapse for cells stimulated as in (B). (D) The proportion of the NK cell synapse predicted to be penetrable by a granule with a diameter of 250 nm for cells stimulated as in (B).

To test whether or not NK cell activation via receptors other than NKG2D similarly remodel the actin cortical mesh, we compared the extent to which cortical F-actin reorganisation occurred in pNK cells following CD16 engagement. pNK cells were stimulated on glass coated with mAb that trigger CD16 (3.0 µg/ml) with and without ICAM-1 (Figure 7A), and again, F-actin was imaged using SI microscopy. The percentage of activated pNK cells following CD16 engagement with or without co-ligation of LFA-1 was equivalent to that observed for NKG2D mediated activation (Figure 7B, 83±2% and 71±2%, respectively, *n* = 30). Importantly, the extent of cortical actin reorganisation was also equivalent, with mean hole areas within the central synapse being 0.26±0.03 µm for cells stimulated on anti-CD16 and 0.19±0.01 µm for cells stimulated with anti-CD16 and ICAM-1 (Figure 7C). In CD16 activated pNK cells stimulated with or without ICAM-1, 4.3±0.3% or 4.0±0.4%, respectively, of the central synaptic region would be penetrable by a granule of diameter 250 nm (Figure 7D). These data demonstrate that similar to NKG2D, CD16-mediated NK cell activation alters the periodicity of the cortical actin mesh which does not require co-ligation of LFA-1.

### F-Actin Organisation Defines Discrete Domains for Lytic Granule Secretion

**Figure 8:**
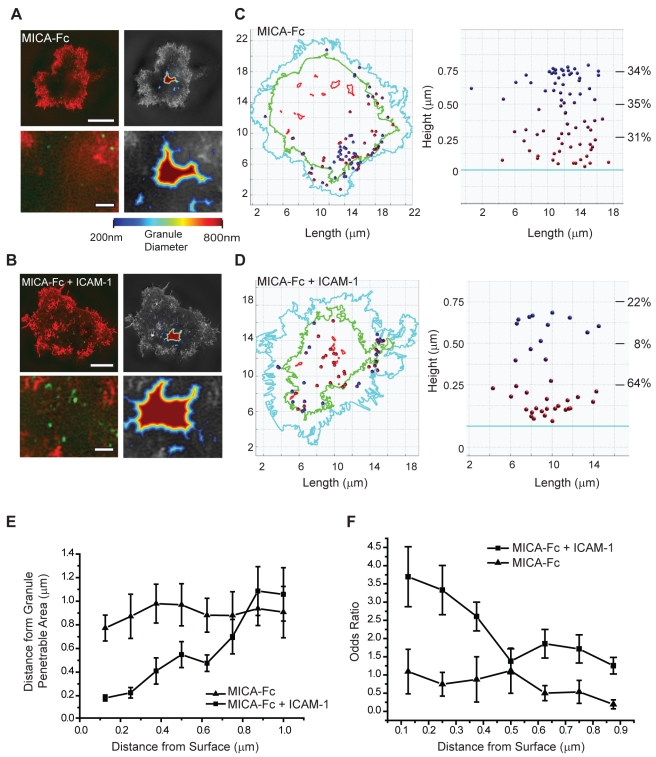
Polarised lytic granules in activated pNK cells preferentially localise to predicted granule penetrative domains. (A) Two-colour SI images of F-actin (red) and perforin (green) (left panels) in the immune synapse of a representative pNK cell activated with MICA-Fc. Right panels show the predicted region of lytic granule penetration for granules with diameter 200–800 nm. Bar = 5 µm. Lower panels show the centre of the synapse enlarged. Bar = 1 µm. (B) As for (A), SI images of F-actin (red) and perforin (green) (left panels) and predicted regions of lytic granule penetration (right panels) for a representative pNK cell activated with MICA-Fc and ICAM-1. Bar = 5 µm. (C) The centroid (position) of each lytic granule was mapped in 3D to a depth of 1 µm above the coverslip, in pNK cells stimulated with MICA-Fc. Left panels show granule positions overlaid onto an *en face* 2D map of the cell-slide contact. This map shows the cell border (cyan), the dense F-actin ring (green), and the regions calculated to be penetrable by granules 200–800 nm in diameter (red). Percentages on right panels represent the proportion of granules which fall within regions 0–250 nm, 250–500 nm, and 500–750 nm of the surface. (D) The centroid for each lytic granule was mapped in 3D as for (C), for pNK cells stimulated with MICA-Fc and ICAM-1. Percentages are shown as in (C). (E) Lateral distances (mean ± SEM) from predicted granule penetrable areas are plotted for granules, within the central synapse, at positions 0–1 µm above the surface. Graph compares pNK cells stimulated with MICA-Fc or MICA-Fc + ICAM-1 (*n* = 10). (F) Calculation of the Odds Ratio for granules to land on granule penetrative areas at axial distances ranging from 0–900 nm above the slide surface that has been coated with MICA-Fc or MICA-Fc + ICAM-1.

To compare the location of lytic granules with domains where the cortical actin mesh had opened up, two-colour 3D-SI microscopy was used to image F-actin and lytic granules together. Primary NK cells were stimulated on slides coated with the NKG2D-ligand MICA either alone or with the LFA-1-ligand ICAM-1 and the cell surface contact area was imaged for F-actin and lytic granules using phalloidin conjugated to ATTO-590 and an anti-perforin mAb directly labelled with Alexa488. In both cases, F-actin accumulated at the synapse periphery and the cortical actin mesh opened up at the synapse centre (Figure 8A and B). Only when NKG2D and LFA-1 were co-ligated were perforin-stained granules polarised to the cell surface. In contrast, when NKG2D was ligated alone, very few granules could be detected at the cell synapse. When NKG2D was ligated alone, the few granules that happened to lie near the contact interface did not co-localise with predicted penetrable domains (Figure 8A, lower panels). Strikingly, when NKG2D and LFA-1 were co-ligated, perforin-stained granules that polarised to the cell surface co-localised with domains where the cortical actin mesh had opened up (Figure 8B, lower panels).

Next, super-resolved z-stacks were taken through the first 1.0 µm into the cell above the contact with the activating surface and images were analysed using a custom MatLab program (see Text S1). For cells stimulated on surfaces coated with MICA-Fc or MICA-Fc and ICAM-1, the position of each granule was mapped onto 2D representation of the cell border, F-actin ring, and domains through which a granule >200 nm in diameter was predicted to pass (Figure 8C and D, Video S4 and S5). Lytic granules in pNK cells stimulated with MICA alone did not localise within the central synaptic region at the cell surface nor co-localised with predicted penetrable domains. Moreover, granules were evenly distributed throughout the first 1 µm up from the cell surface (Figure 8C right hand panel). In contrast, in cells stimulated with MICA and ICAM-1, two-thirds of lytic granules were located within 250 nm of the surface, i.e. were likely docked with the synaptic membrane (Figure 8D right panel). Importantly, lytic granules docked at domains where the cortical actin mesh had increased periodicity (Figure 8D left panel, compare the location of red lytic granules with the red line marking granule penetrable domains). Thus, lytic granules specifically dock where the cortical actin mesh has opened up.

To analyse the location of lytic granules across many cells, the lateral distance from a penetrable area was determined for each lytic granule within 1 µm up from the contact with the glass slide. For each granule at a given height above the surface, the lateral but not the axial distance from predicted granule penetrative domains was measured and plotted against the distance of that granule from the contact with the glass surface. This analysis revealed that when NKG2D and LFA-1 were co-ligated, granules within 400 nm of the cell surface interface polarised to within a few hundred nanometers of predicted penetrable areas, whereas granules >500 nm above the cell surface or granules in cells for which LFA-1 was not co-ligated tended to localise >1 µm in the lateral direction away from predicted penetrable domains (Figure 8E). As an alternative analysis, the tendency of lytic granules to localise within domains where the cortical actin mesh opened up was calculated as an Odds Ratio, defined as:
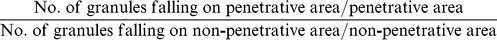
This analysis revealed that lytic granules within 400 nm of the interface in pNK cells stimulated through NKG2D and LFA-1 were located within the regions of cortical actin which had opened up the most (indicated by odds ratios between 2.5 and 3.75, Figure 8F). This is particularly striking as only ∼5% of the central synapse was predicted to be penetrable by granules of this size. Thus, for pNK cells in which NKG2D and LFA-1 are ligated, polarised lytic granules localise to the specific domains in the synapse centre where the cortical actin structure has opened up to permit granules to pass through.

### The MTOC Polarises to Secretory Domains Defined by F-Actin Structure

**Figure 9:**
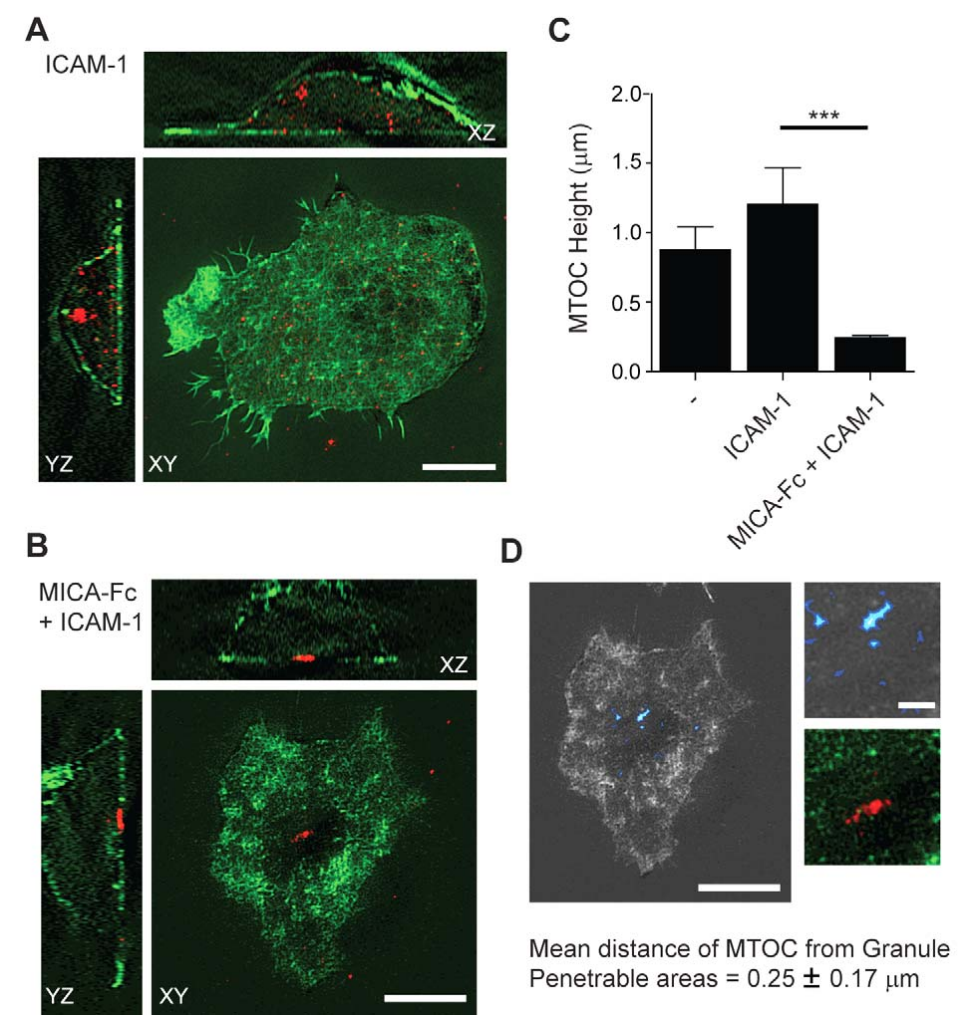
MTOC polarisation towards Granule Penetrable Areas. (A) Two-colour 3D-SI image of F-actin (green) and the MTOC (red) in a pNK cell stimulated on an ICAM-1 coated surface. Upper and left panels show orthogonal XZ and YZ slices, respectively, taken from the super-resolved Z-stack. (B) Two-colour 3D-SI image of F-actin (green) and the MTOC (red) in a pNK cell stimulated on a surface coated with MICA-Fc and ICAM-1. Upper and left panels show orthogonal XZ and YZ views of the super-resolved Z-stack, respectively. (C) The average axial distance or “height” of the MTOC above the cell-surface interface for pNK cells on poly lysine, ICAM-1, or MICA-FC + ICAM-1 coated surfaces. Graph shows mean ± SD (*n* = 10 cells per condition (*** *p*<0.001). (D) Left and upper right panels show the regions of the cortical F-actin mesh through which a lytic granule diameter of 200–800 nm in diameter would be predicted to pass. Lower right panel shows the presence of the MTOC at the cell surface interface, in close proximity to the granule penetrable areas in a pNK cell stimulated on MICA-Fc + ICAM-1. The distance of the MTOC from granule penetrable areas for *n* = 7 cells is 0.25±0.17 µm (mean ± SD). Scale bars = 5 µm except right panels in (D) where scale bars = 1 µm.

We have shown that co-ligation of LFA-1 is essential to NKG2D-mediated polarisation of granules to predicted penetrable domains within the synapse. As it had been previously demonstrated that ligation of LFA-1 is required for directed granule delivery to the NK cell immune synapse via the polarisation of the MTOC, we next investigated whether the MTOC polarises towards predicted domains of granule secretion in the F-actin structure at the NK cell synapse. pNK cells were incubated on glass coated with poly-L-lysine, ICAM-1, or MICA-Fc with ICAM-1 and two-colour 3D-SI microscopy was used to determine the relative location of the MTOC and domains where the periodicity of the actin mesh had opened up (Figure 9A and B). The MTOC was observed to reorientate to within a few hundred nanometers of the cell-surface interface (0.24±0.07 µm, *n* = 15) when NKG2D and LFA-1 were co-ligated, but did not polarise when cells were unstimulated (0.87±0.4 µm) or LFA-1 alone was ligated through ICAM-1 (1.2±0.74 µm). Strikingly, the polarised MTOC in cells stimulated via NKG2D and LFA-1 was consistently located in close proximity to regions of the synapse easily penetrable by granules of 250 nm in diameter (mean lateral distance, 0.25±0.17 µm, *n* = 8 cells). The fact that the cortical actin mesh opens up even when integrins are not engaged and polarisation of the MTOC is not triggered would suggest that this event occurs independently, and perhaps initially. Taken together this indicates a model of secretion in which polarisation of the MTOC delivers lytic granules to specific regions where the actin mesh has opened up.

## Discussion

NK cell cytotoxic function is essential for the elimination of virally infected and tumour cells and is regulated through the rearrangement of receptors, signalling molecules, and the cytoskeleton at the immune synapse. A major barrier to visualising molecular events that control NK cytotoxicity at the intercellular synapse has been the resolution limit of conventional light microscopy. This has made it difficult to study many key steps in the cytolytic process including the dynamic organisation of protein microclusters and the organisation of the actin cytoskeleton known to be important for degranulation. Here, we have addressed these unknowns using two methods for imaging the NK cell cytolytic synapse with unprecedented spatial resolution. Firstly, optical trapping technology allowed us to follow the organisation of receptor microclusters at the intercellular contact revealing that microclusters move centripetally to form a ring-shaped structure at the synapse centre. Secondly, we used super-resolution imaging to define the F-actin structure within this central synaptic region and show that synaptic remodelling of F-actin creates discrete domains where the MTOC polarises and lytic granules dock.

Previously the organisation of ICAM-1 and the NKG2D ligand ULBP1 in a central synaptic ring has been shown to mark out the location of bi-directional vesicle trafficking [31]. Building on these observations, we show here that NK cell synapse formation is rapid after intercellular contact such that microclusters of NKG2D assemble on a sub-second timescale and move centripetally to form a stable ring-shaped structure within minutes. Recruitment of signalling molecules Grb2 and Vav1 was sustained throughout the rearrangement of NKG2D microclusters into a central synaptic ring. The spatial reorganisation of NKG2D microclusters into a stable synaptic ring occurred where lytic granules polarised to the synapse centre. Rings of NKG2D and signalling molecules formed at the intercellular synapse within a peripheral actin ring. It remains to be tested whether or not the ring-shaped organisation of NKG2D is directly involved in establishing the secretory domain.

This is reminiscent of the formation of an organised CTL synapse that increases the efficiency of both granule polarisation and target cell killing [24],[59]–[60]. In contrast to T cells, NK cell cytotoxicity requires signals from multiple activating receptors which are integrated in order to stimulate both granule polarisation and degranulation [10]. Engagement of LFA-1 is sufficient to induce granule polarisation in NK cells but co-ligation with activating receptors is required for directed degranulation [61]. This has led to the suggestion that NK cell cytotoxicity is a step wise process with multiple “checkpoints” [13],[62]–[64]. One well-established step in the formation of the cytolytic synapse following activating receptor ligation is the organisation of F-actin in a distinct ring at the synapse periphery [14],[36]. It has previously been suggested that concurrent to forming this peripheral ring, F-actin is entirely cleared from the synapse centre to allow lytic granule secretion. This has been suggested for both CTL [20] and NK cell synapses [14],[33].

Our data show that this model needs some modification because cortical actin is not entirely cleared from the synapse centre. Instead, NK cell activation, through NKG2D or CD16 (and likely other NK cell activating receptors), remodels the cortical actin structure at the synapse centre and is not dependent on co-ligation of LFA-1. This leads to opening of the cortical actin mesh to produce discrete domains within the central region of the synapse sufficient for individual lytic granules, shown here to be ∼250 nm in diameter, to pass through relatively unimpeded. Importantly, these discrete gaps are commonly juxtaposed, not merely scattered homogenously across the synapse, consistent with the formation of specific secretory domains. Crucially, while such granule penetrable domains made up only approximately 5% of the synapse, we found that the MTOC reorientated towards these domains and polarised lytic granules docked precisely at these regions. In NK cells at least, remodelling of cortical F-actin may be important to allow lytic granule secretion.

In unactivated NK cells, cortical actin within the centre of the immune synapse may present a barrier for lytic granule secretion, as suggested for other types of secretion [65]. For example, in pancreatic β cells, the cortical actin structure has been shown to act as a barrier in the regulation of vesicle exocytosis [66]. Similarly here we found that unactivated primary human NK cells have a dense cortical actin structure that is predicted to be impenetrable by lytic granules >100 nm in diameter and is therefore likely to require some degree of remodelling to permit granule secretion.

In many other cell types there is evidence that remodelling of dense cortical actin regulates granule secretion. Granule exocytosis in mast cells is reliant on both actin polymerisation and the remodelling of cortical actin to allow granules access to the plasma membrane [67]. In mast cells these processes are thought to be controlled by independent signalling pathways, with cortical actin remodelling dependent on the p21-activated kinase, Pak1 [68]. Furthermore, in neutrophils, Rac-dependent cortical actin remodelling mediates granule exocytosis [69]. Interestingly in NK cells, both Pak1 and Rac pathways are activated following ligation of NKG2D or CD16 and the recruitment of Vav-1 [50],[52],[70]. Thus, it would be interesting to establish if remodeling of cortical actin within the central region of the NK cell synapse is also controlled by this pathway [51]. Myosin IIA or other granule associated proteins could mechanically facilitate granule motility through the actin structure, similar to the release of catecholamine granules from chromaffin cells [71], or may be involved in inducing local changes in actin structure to allow granules to pass through the F-actin once they arrive at the synapse. It is intriguing that polarisation of the MTOC and lytic granules is dependent on LFA-1 engagement while actin rearrangement can be triggered independently of LFA-1. This suggests that F-actin remodelling is independent of granule polarisation and may therefore happen first. If this is the case, an interesting new goal is to understand how the MTOC brings granules specifically to where the actin mesh has opened up and how this is regulated by a LFA-dependent signal. In summary, our data define remodelling of cortical actin at the central region of the synapse as a new aspect of the process involved in secretion of lytic granules across an immune synapse.

## Materials and Methods

### Cell Lines and Primary NK Cells

NKL were maintained in RPMI-1640 supplemented with 10% FCS, 100 µg/ml streptomycin, 100 µg/ml penicillin, 100 µg/ml L-glutamine, and 100 U/ml IL-2 (all Invitrogen; complete media). Daudi/MICA cell lines were cultured in complete medium supplemented with 20% FCS (Invitrogen). NKL transfected to express actin-YFP were obtained and cultured as described previously [55]. NKL transfected to express NKG2D-GFP (NKL/NKG2D-GFP) or Vav1-GFP and Grb2-mCherry (NKL/Vav1-GFP/Grb2-mCherry) were generated by retroviral transfection of NKL cells as described [48]. Briefly, the packaging cell line Phoenix was transfected with PINCO-GFP-NKG2D, or PINCO-Vav1-GFP then PINCO-Grb2-mCherry plasmid using lipofectamine LTX (Invitrogen). Viral supernatant collected 24 and 48 h post-transfection were used for three sequential centrifugations (45 min, 725× g) for infection of 5×10^5^ NKL cells. After 1 wk, cells expressing GFP, or GFP and mCherry were selected by flow cytometry. NK cell cytotoxicity against different target cells was assessed in standard ^35^S-Methionine release assays performed in triplicate. For CD16 mediated cytotoxicity Daudi target cells were first labelled with 0.1 µg anti-CD20, for 15 min, at 37°C.

Primary human NK cells were isolated from healthy donor peripheral blood under negative magnetic selection (NK cell isolation kit; Miltenyi Biotec) and cultured as previously described [72]. Freshly isolated NK cells were stimulated with 150 U/ml human recombinant IL-2 (Roche) and experiments were carried out 6 d later.

### Plasmid Generation

The plasmid containing Vav1-GFP was generated previously [48]. The coding sequence for NKG2D was amplified from human complementary cDNA (cDNA) using the primers: forward 5′-CGTGTACAAGGGAGGCGGTTCAGGTGGAGGCTCGATGGGGTGGATTCGT-3′ and reverse 5′-CGCTCGAGCGGCCGCTCTACACAGTCCTTTGCATGC-3′. NKG2D cDNA was then N-terminally tagged with GFP by subcloning into pcDNA3.1 (Invitrogen) encoding eGFP using BsrgI/NotI restriction sites. NKG2D-GFP was then sub-cloned as a BamHI/NotI fragment into the retroviral PINCO vector [73]. The coding sequence for Grb2 was amplified from human complementary cDNA using the primers: forward 5′-CGGAATTCATGGAAGCCATCGCCAAATATGAC-3′ and reverse 5′-CGGGATCCGAGCCTCCACCTGAACCGCCTCCGACGTTCCGGTTCACGGG-3′. Grb2 cDNA was C-terminally tagged with mCherry by subcloning into pEGFPN1 (Clontech) vector in which GFP had been exchanged for mCherry (pmCherryN1). Grb2-mCherry was then sub-cloned as a BamHI/NotI fragment into the retroviral PINCO vector.

### Confocal Microscopy Using Optical Tweezers

NKL/NKG2D-GFP and NKL/Vav1-GFP/Grb2-mCherry transfectants were mixed in a 1∶1 ratio with Daudi/MICA cells in chamber slides (Nunc) that had been blocked with 100% FCS. For staining of lytic granules NKL/NKG2D-GFP cells were incubated with 100 nM Lysotracker Red (Molecular Probes) for 2 h before imaging. For fixed cell staining, NKL/NKG2D-GFP and Daudi/MICA cells were mixed in a 1∶1 ratio for 12 min. Conjugates were fixed in 4% paraformaldehyde and permeabilised with Triton X-100 (Sigma) before staining with anti-perforin mAb (clone δG9, BD Biosciences) followed by Alexa633-conjugated goat anti-mouse IgG mAb (Molecular Probes) at room temperature for 45 min. High speed, high resolution live cell imaging of the forming synapse between the NKL cell and target cell was carried out using optical tweezers as previously described [32]. Briefly, a single spot E3300 commercial optical tweezer generated by a 1070 nm solid state laser (Elliot Scientific UK) was coupled into a commercial confocal microscope (TCS SP5, Leica Microsystems Ltd). Target cells were optically trapped and placed onto the target cell using the tweezer and images were acquired at a rate of 1 fps using a 63×, 1.25 NA water-immersion objective. Simultaneous imaging of multiple fluorophores was achieved by sequential line scanning. Image analysis was carried out using ImageJ software (NIH).

### Preparation of Coated Slides

Glass coverslips were prepared as previously described [55]. Briefly, slides were cleaned using 70% ethanol, coated with 0.01% poly-L-lysine, dried, and then coated with mAb or recombinant proteins in PBS. Slides were then washed and blocked with complete medium. Antibodies to NKG2D (Clone 149810, R&D systems, 5.0 µg/ml), NKG2A (Clone 131411, R&D systems, 5.0 µg/ml) or CD16 (Clone 3G8 BD Pharmingen, 5.0 µg/ml) or murine IgG isotype controls (Jackson Immunology), recombinant MICA-Fc (R&D systems, 2.0 µg/ml), or recombinant ICAM-1 (R&D systems, 2.5 µg/ml) were used.

### Antibody Blocking

For blocking of NKG2D or CD16-mediated activation of NK cells, pNK cells were incubated for 15 min at 37°C with antibodies to NKG2D or CD16 (5.0 µg/ml) or human recombinant MICA (Autogen Bioclear) before incubation on coated surfaces.

### 3D-Structured Illumination Microscopy

NKL or primary NK cells were added in complete media supplemented with 25 mM HEPES to antibody- or ligand-coated slides, then fixed after 6 min in 4% PFA and permeabilised with 0.1% TritonX-100. To visualise F-actin, cells were stained with 2 U/ml phalloidin-AlexaFluor 488 (Invitrogen). To visualise lytic granules, cells were stained with AlexaFluor 488 labelled anti-perforin mAb (BD Pharmingen), and to visualise the MTOC, cells were fixed in MeOH and stained with rabbit anti-γ tubulin (Clone T5192 Sigma) followed by secondary detection with AlexaFluor 594 conjugated goat anti-rabbit antibody (Molecular Probes). The presence of an actin ring was defined as a broad ring of intense F-actin staining surrounding a cell centre with low F-actin fluorescence, as previously described [55]. Brightfield, fluorescence, and IRM images were obtained using a confocal microscope (Leica SP5 RS) with a 63× water immersion lens (NA 1.2).

Super-resolution images of fixed samples or 40 nm beads were acquired by 3D-Structured illumination microscopy [74] using an OMX microscope (Applied Precision) and 100×, 1.4 NA oil objective (Olympus) as previously described [75].

### Image Analysis

Structured illumination images were analyzed with a custom-written Matlab (Mathworks) program (Text S1). The size of actin rings was obtained using ImageJ (NIH). For the determination of Lysotracker localization at the immune synapse relative to the NKG2D ring, the regions defined by the inner and outer borders of the ring were overlayed on the Lysotracker images and Lysotracker areas quantified in and outside these domains using the particle analysis tool in ImageJ.

### Statistical Analysis

Column Statistics were performed with GraphPad software (Prism). Pearson's correlation coefficients (R_r_) were calculated by intensity correlation analysis with ImageJ (NIH). Mean values are shown. Errors and error bars represent SEM unless otherwise stated. In statistical analysis, *p* values >0.05 are indicated as not significant (NS), and *p* values <0.001 are indicated by three asterisks (***). In Figure 7 the Odds Ratio was calculated by:
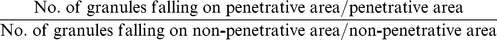



## Supporting Information

Figure S1 NKL transfected with NKG2D-GFP effectively kill Daudi/MICA. (A) Untransfected NKL and NKL/NKG2D-GFP cells were tested for their ability to lyse Daudi/MICA target cells at different E∶T ratios. (B) The specificity of NKL/NKG2D-GFP target cell lysis was tested by their ability to lyse Daudi/MICA or Daudi cells in which MHC class I expression has been rescued by transfection of β2-microglobulin (Daudi/β2M). Data are representative of three independent experiments performed in triplicate; graphs show mean ± SEM (*n* = 3).
(TIF)

Figure S2 Dynamic reorganisation of Vav1-GFP at the NKL cell synapse. (A) Time-lapse imaging (0.9 fps) of Vav1-GFP microcluster formation and reorganisation into a ring-shaped structure between NKL expressing Vav1-GFP and Grb2-mCherry and Daudi/MICA. (B) Time-lapse imaging (1 fps) showing Vav1-GFP or Grb2-mCherry did not accumulate when NKL expressing Vav1-GFP and Grb2-mCherry were brought into contact with Daudi cells that did not express MICA. Bars = 5 µm.
(TIF)

Figure S3 Sizing of the actin ring formed in activated NK cells. (A) The diameter of the inner and outer borders of F-actin rings formed in NKL cells stimulated on surfaces coated with MICA-Fc with or without ICAM-1 or NKL cells expressing actin-YFP in conjugates with Daudi/MICA. Graph shows mean ± SD (*n* = 10).
(TIF)

Figure S4 CD16 does not contribute to NK cell activation on NKG2D-coated slides. (A) The mAb to CD16 (3G8) was demonstrated to block CD16-specific lysis by pNK cells of Daudi/β2M or Daudi/β2M/MICA coated with anti-CD20. Data are representative of three independent donors, with experiments performed in triplicate. Graph shows mean ± SEM. (B) The proportion of pNK cells activated on poly lysine, αNKG2D, or MICA-Fc and ICAM-1 coated slides following pre-incubation of the cells with either αNKG2D, αCD16 (3G8), or MICA ligand. Graph shows mean ± SEM (*n*>50 cells per condition).
(TIF)

Text S1 Structured Illumination image analysis with a custom-written MatLab program.
(DOC)

Video S1 NKG2D organises into a central ring during NK cell immune synapse formation. Live cell imaging of NKG2D microcluster formation and organisation into a central synaptic ring when an NKL/NKG2D-GFP was contacted to a Daudi/MICA. Cells were imaged at a frame rate of 1 fps and are shown at 10 fps. Bar = 5 µm.
(MOV)

Video S2 Lytic granules are not polarised during NKG2D microcluster formation. Live cell imaging of the early synapse formed between NKL/NKG2D-GFP loaded with Lysotracker red and Daudi/MICA. The movie shows that during formation of NKG2D microclusters Lysotracker does not significantly polarise. Cells were imaged at a frame rate of 1 fps for 60 s and are shown at 7 fps. Bar = 4 µm.
(MOV)

Video S3 Lytic granules polarise within the NKG2D ring at the NK cell immune synapse. Live cell imaging of the same conjugate shown in Video S2 imaged later, after organisation of NKG2D microclusters into a central synaptic ring. Lysotracker is now polarised to the central synapse within the ring-shaped organisation of NKG2D. Cells were imaged at 1 fps and are shown at 7 fps. Bar = 4 µm.
(MOV)

Video S4 Lytic granule organisation at the immune synapse in NK cells activated with MICA. 3D reconstruction of lytic granule centroids localised within 1 µm of the cell surface in pNK cells activated on coverslips with MICA-Fc. Centroids are mapped onto a 2D image showing the cell borders (cyan), the dense actin ring (green), and predicted granule penetrable areas (red).
(MOV)

Video S5 Lytic granule organisation at the immune synapse in NK cells activated with MICA and ICAM-1. 3D reconstruction as in Video S4, but for pNK cells stimulated on coverslips coated with MICA-Fc and ICAM-1.
(MOV)

## Abbreviations

3D-SI microscopy: 3D-Structured Illumination microscopy
CTLs: Cytotoxic T lymphocytes
F-actin: Filamentous actin
FWHM: Full width-half maximum
IL-2: Interleukin-2
MTOC: Microtubule-organising centre
NK cell: Natural Killer cell
pNK: Primary NK
TIRF microscopy: Total Internal Reflection Fluorescence microscopy
WASp: Wiskott-Aldrich Syndrome protein
