# Correction: Remodelling of Cortical Actin Where Lytic Granules Dock at Natural Killer Cell Immune Synapses Revealed by Super-Resolution Microscopy

**DOI:** 10.1371/annotation/cfe6f47e-8c81-4ef0-bdf3-8841cbe40b93

**Published:** 2012-08-30

**Authors:** Alice C. N. Brown, Stephane Oddos, Ian M. Dobbie, Juha-Matti Alakoskela, Richard M. Parton, Philipp Eissmann, Mark A. A. Neil, Christopher Dunsby, Paul M. W. French, Ilan Davis, Daniel M. Davis

The authors inadvertently duplicated two of the lower-panel “zoomed in” images in Figure 4D. The correct Figure 4D is here: 

**Figure:**
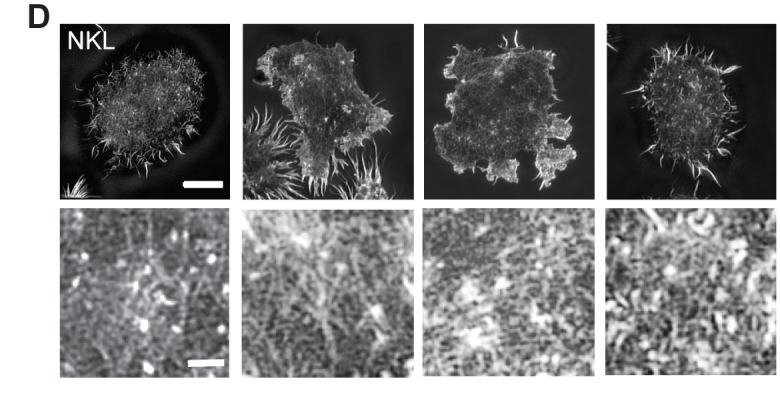


The full correct Figure 4 is here: 

**Figure:**
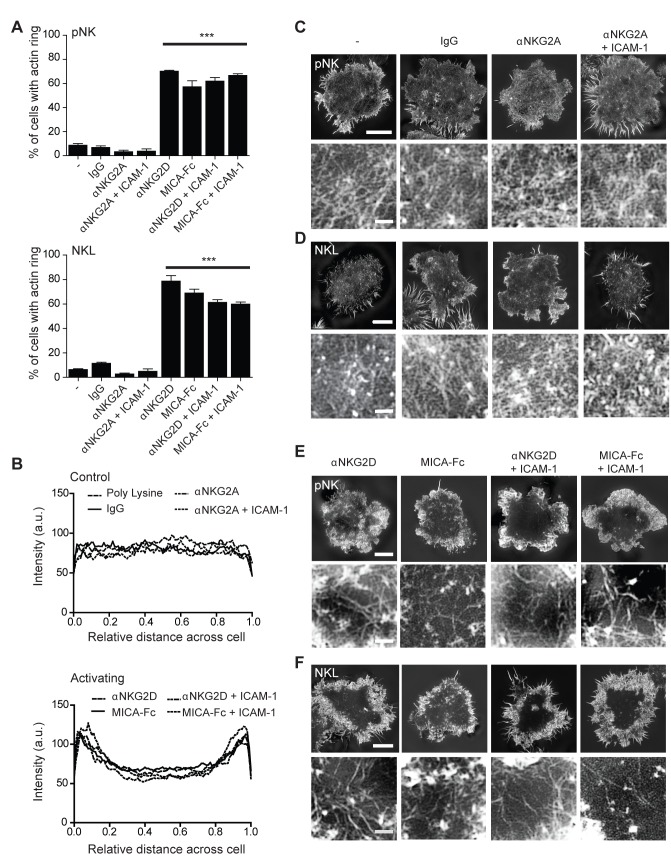


The authors also wish to clarify that the representative images used in this figure were intentionally used in Figures 5 and 6 so that the reader could follow how they first imaged the cells (Fig. 4), then analysed the holes within the actin mesh (Fig. 5), then analysed the penetrable areas within the actin mesh (Fig. 6). To clarify this, the final sentence of the legend to Figure 4 should read, “The representative SI images of pNK cells were used in subsequent Figures 5 and 6 to clearly demonstrate how computational analysis was sequentially applied to each super-resolution image in order to quantify changes in actin mesh structure.” In Figures 4, 5, and 6, the n numbers were given in summary as n = 10–75 cells per condition. In full detail, n = 10 for cells stimulated on poly lysine, n = 50 for IgG, n = 58 for αNKG2A, n = 51 for αNKG2A + ICAM1, n = 60 for ICAM-1, n = 62 for αNKG2D, n = 68 for MICA-Fc, and n = 75 for αNKG2D + ICAM-1 or MICA-Fc + ICAM-1.

