# Supplementary figures and images for: Remodelling of Cortical Actin Where Lytic Granules Dock at Natural Killer Cell Immune Synapses Revealed by Super-Resolution Microscopy
Source: PLoS Biol. 2011 Sep 13;9(9):e1001152. doi: 10.1371/journal.pbio.1001152 (PMC3172219; doi:10.1371/journal.pbio.1001152)

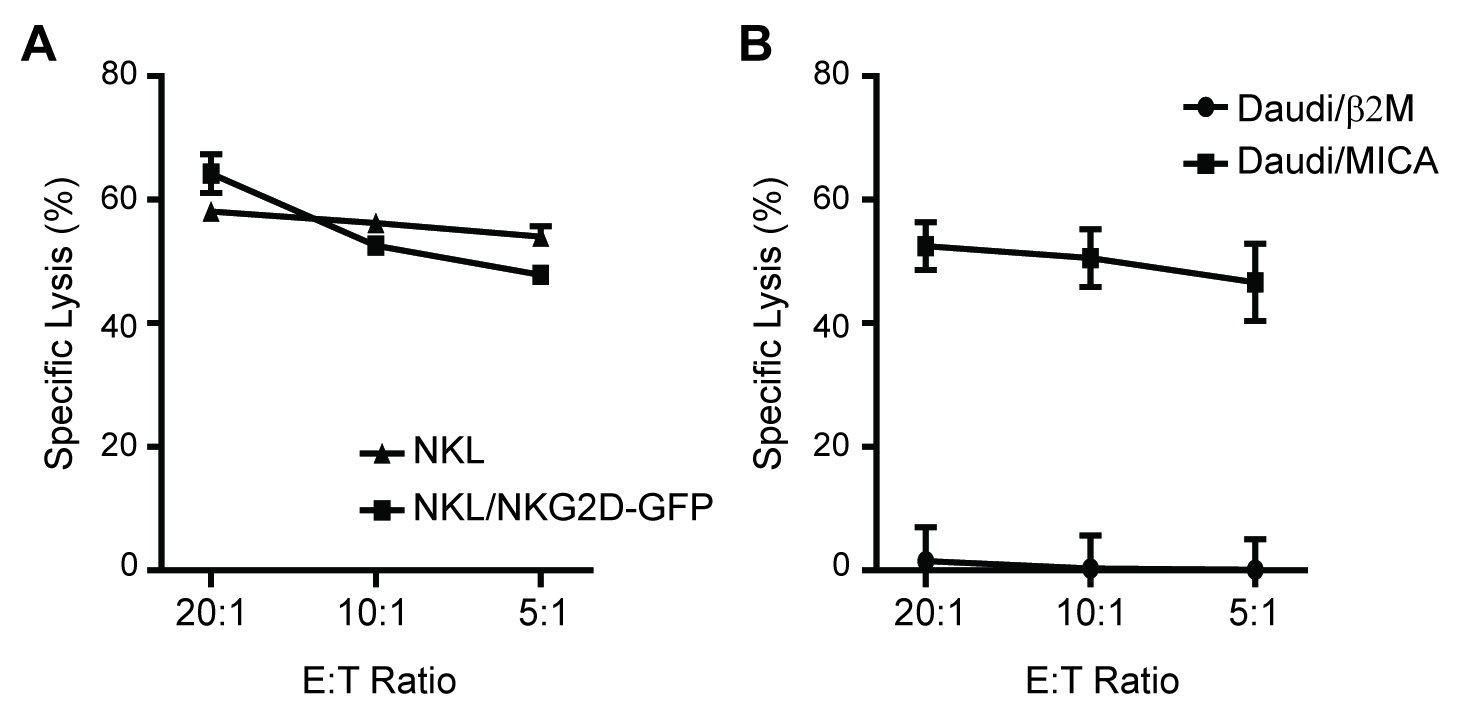

Supplement: Figure S1 — NKL transfected with NKG2D-GFP effectively kill Daudi/MICA. (A) Untransfected NKL and NKL/NKG2D-GFP cells were tested for their ability to lyse Daudi/MICA target cells at different E∶T ratios. (B) The specificity of NKL/NKG2D-GFP target cell lysis was tested by their ability to lyse Daudi/MICA or Daudi cells in which MHC class I expression has been rescued by transfection of β2-microglobulin (Daudi/β2M). Data are representative of three independent experiments performed in triplicate; graphs show mean ± SEM (n = 3). (TIF) [file pbio.1001152.s001.tif]

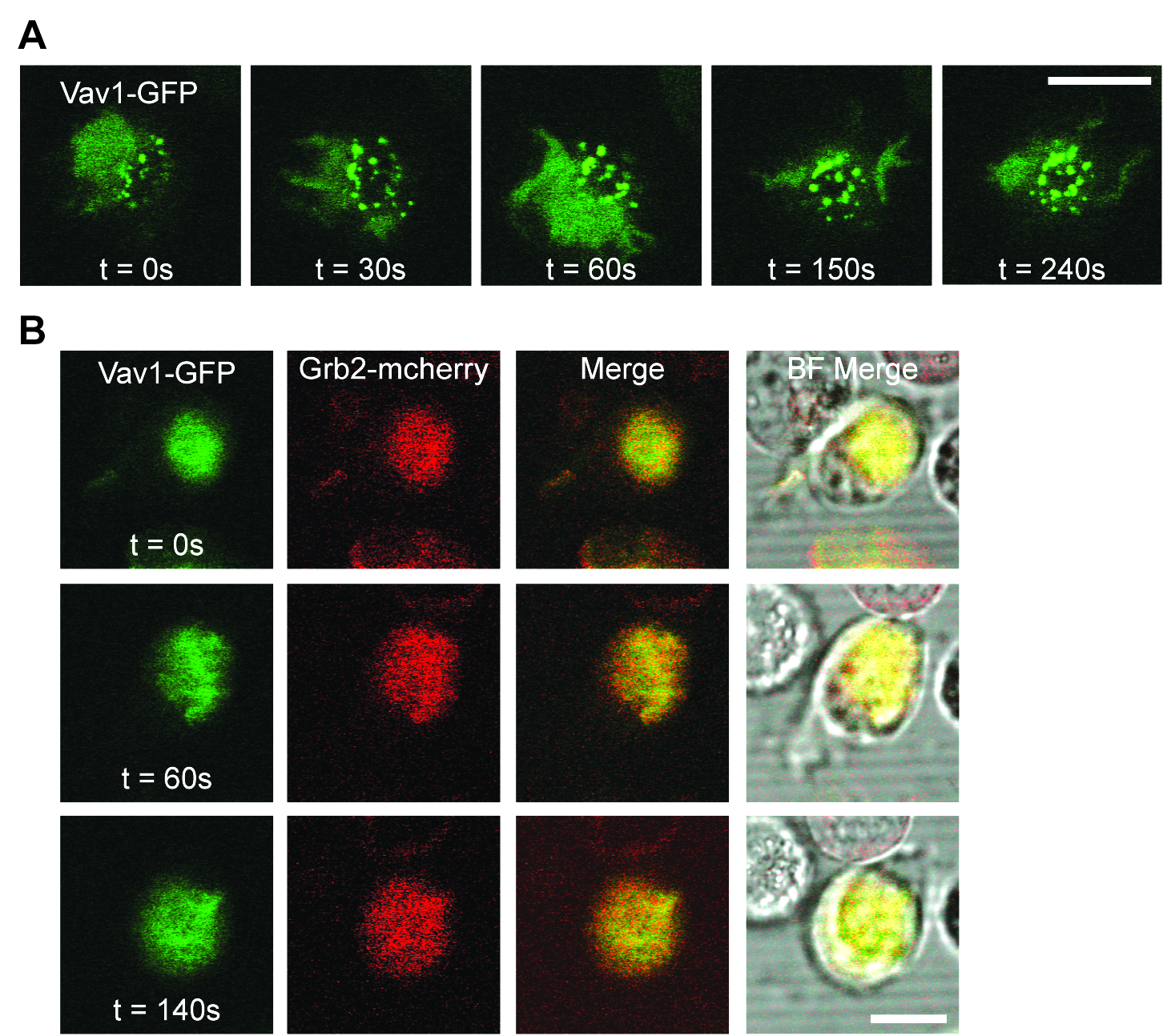

Supplement: Figure S2 — Dynamic reorganisation of Vav1-GFP at the NKL cell synapse. (A) Time-lapse imaging (0.9 fps) of Vav1-GFP microcluster formation and reorganisation into a ring-shaped structure between NKL expressing Vav1-GFP and Grb2-mCherry and Daudi/MICA. (B) Time-lapse imaging (1 fps) showing Vav1-GFP or Grb2-mCherry did not accumulate when NKL expressing Vav1-GFP and Grb2-mCherry were brought into contact with Daudi cells that did not express MICA. Bars = 5 µm. (TIF) [file pbio.1001152.s002.tif]

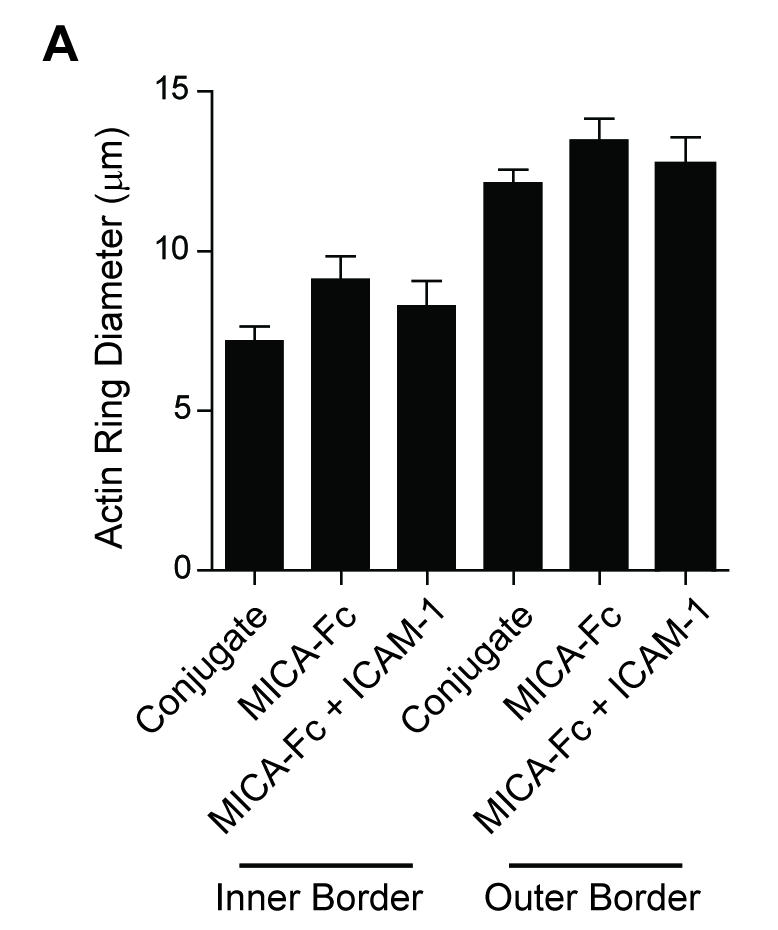

Supplement: Figure S3 — Sizing of the actin ring formed in activated NK cells. (A) The diameter of the inner and outer borders of F-actin rings formed in NKL cells stimulated on surfaces coated with MICA-Fc with or without ICAM-1 or NKL cells expressing actin-YFP in conjugates with Daudi/MICA. Graph shows mean ± SD (n = 10). (TIF) [file pbio.1001152.s003.tif]

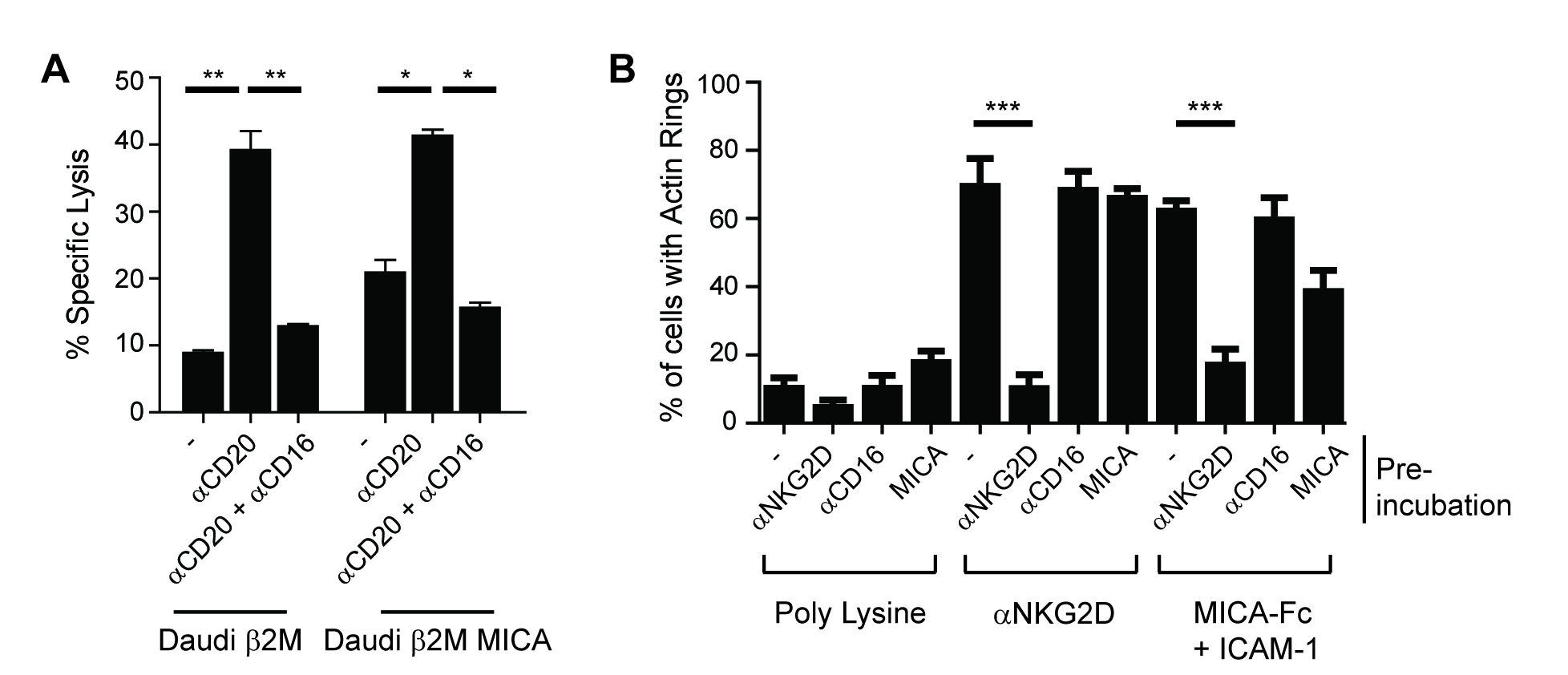

Supplement: Figure S4 — CD16 does not contribute to NK cell activation on NKG2D-coated slides. (A) The mAb to CD16 (3G8) was demonstrated to block CD16-specific lysis by pNK cells of Daudi/β2M or Daudi/β2M/MICA coated with anti-CD20. Data are representative of three independent donors, with experiments performed in triplicate. Graph shows mean ± SEM. (B) The proportion of pNK cells activated on poly lysine, αNKG2D, or MICA-Fc and ICAM-1 coated slides following pre-incubation of the cells with either αNKG2D, αCD16 (3G8), or MICA ligand. Graph shows mean ± SEM (n>50 cells per condition). (TIF) [file pbio.1001152.s004.tif]
