# Supplementary material for: Remodelling of Cortical Actin Where Lytic Granules Dock at Natural Killer Cell Immune Synapses Revealed by Super-Resolution Microscopy
Source: PLoS Biol. 2011 Sep 13;9(9):e1001152. doi: 10.1371/journal.pbio.1001152 (PMC3172219; doi:10.1371/journal.pbio.1001152)
Supplement: Text S1 — Structured Illumination image analysis with a custom-written MatLab program. (DOC) [file pbio.1001152.s005.doc]

**Supporting Information**

**Text S1: Image Analysis**

Structured illumination images were analysed with a custom-written MatLab (Mathworks) program. A region containing a cell of interest was first cropped manually, and then an empty region of background was selected to evaluate the background intensity characteristics. For the purpose of finding the borders, the mean background intensity was subtracted from the image, and the borders of the cell and the central synaptic zone inside the dense peripheral actin ring were found using Chunming Li’s region scalable fitting energy minimization algorithm [1] and corrected manually if necessary. For analysis of stacks containing several optical slices in z, the algorithm was run for the slices individually, as this proved faster than the 3D version of this algorithm without significant differences in the quality of the results. The intensity characteristics for different regions were then calculated from the unmodified cropped image or stacks. To obtain the profile of actin intensity across a cell, a line was manually drawn and at each position intensities along the line normal in the xy plane were integrated over a total width of eight pixels (four on both sides of the line). The narrow actin filaments of the dense actin mesh, crossed at sharp angles, and had strongly variable background intensity, and proved difficult to identify using level set-based methods. Instead thresholding with extensive preprocessing was used. As the resolution-limited actin filaments were ~3 pixels wide, first an ordinance filter in a 5-by-5 neighborhood was used to find the local minimum intensity. To avoid pixelation, the local minimum image was convolved with a Gaussian function (σ = 5 pixels) using a fifteen pixels wide kernel. The convolved minimum intensity image was subtracted from the cropped, unmodified image. The resulting image was rescaled from 0 to 1 and the borders in the image were deblurred by deconvolution with a Gaussian function (with σ=0.8 pixels and kernel width = 5 pixels) using the Lucy­­–Richardson algorithm [2,3]. For the actin staining channel Otsu’s method was then used for a region limited to pixels inside the cell to estimate the grey level threshold [4]. A multiplier for the threshold was then modified manually while comparing the thresholded image to the original image. We confirmed this process removed low signal artifacts that might be present in the background of reconstructed images by processing reconstructed Bead images in which reconstruction artifacts were clearly visible. This showed that only true bead fluorescence and no reconstruction artifacts were detected post processing.

The granule channel contained hardly any background intensity anywhere in the image, so a simpler approach was used. The image was smoothed with Damien Garcia’s algorithm [5]. The local minimum convolved with Gaussian was then subtracted as above. After this, the values for 90^th^ percentile background intensity and the mean background intensity + 2×standard deviation were calculated, and the maximum of these values was subtracted from the image. After this, Otsu gray level threshold for the pixels inside the image was computed, and this value was multiplied by 0.25 before thresholding, this ensured that all granules were found.

After thresholding, the areas covered by actin as well as the sizes of holes in the actin mesh were calculated for the surface slice, and a distance transform was used to calculate the minimum distance to an actin-positive pixel for all pixels within the cell. This distance transform image was then used to generate the granule-penetrable area image and to calculate the fraction of granule penetrable area for different granule diameters by selecting the pixels whose distance to the closest actin positive pixel was more than half the granule diameter. The granules were simply taken as connected regions in 3D, centroids for these regions were obtained using standard Matlab functions, and the granule volume was taken as volume of the pixels of these regions. The granule surface area was obtained by calculating the isosurface triangulation, multiplying the X, Y, and Z pixel coordinates by the respective pixel dimensions, and summing the areas of the triangles individually for each granule.

**Supporting References**

1. Li C, Kao CY, Gore JC, Ding Z (2008) Minimization of region-scalable fitting energy for image segmentation. IEEE Trans Image Process 17: 1940-1949.

2. Richards.Wh (1972) Bayesian-Based Iterative Method of Image Restoration. Journal of the Optical Society of America 62: 55-&.

3. Lucy LB (1974) Iterative Technique for Rectification of Observed Distributions. Astronomical Journal 79: 745-754.

4. Otsu N (1979) Threshold Selection Method from Gray-Level Histograms. Ieee Transactions on Systems Man and Cybernetics 9: 62-66.

5. Garcia D (2010) Robust smoothing of gridded data in one and higher dimensions with missing values. Computational Statistics & Data Analysis 54: 1167-1178.
